# Combinations of Piperine with Hydroxypropyl-β-Cyclodextrin as a Multifunctional System

**DOI:** 10.3390/ijms22084195

**Published:** 2021-04-18

**Authors:** Anna Stasiłowicz, Natalia Rosiak, Ewa Tykarska, Maciej Kozak, Jacek Jenczyk, Piotr Szulc, Joanna Kobus-Cisowska, Kornelia Lewandowska, Anita Płazińska, Wojciech Płaziński, Judyta Cielecka-Piontek

**Affiliations:** 1 Department of Pharmacognosy, Faculty of Pharmacy, Poznan University of Medical Sciences, Swiecickiego 4, 60-781 Poznań, Poland; astasilowicz@ump.edu.pl (A.S.); nrosiak@ump.edu.pl (N.R.); 2 Department of Chemical Technology of Drugs, Poznan University of Medical Sciences, Grunwaldzka 6, 60-780 Poznan, Poland; etykarsk@ump.edu.pl; 3 Department of Macromolecular Physics, Faculty of Physics, Adam Mickiewicz University, Uniwersytetu Poznańskiego 2, 61-614 Poznań, Poland; mkozak@amu.edu.pl; 4 NanoBioMedical Centre, Adam Mickiewicz University, Wszechnicy Piastowskiej 3, 61-614 Poznań, Poland; jacek.jenczyk@amu.edu.pl; 5 Department of Agronomy, Poznań University of Life Sciences, Dojazd 11, 60-632 Poznań, Poland; piotr.szulc@up.poznan.pl; 6 Department of Gastronomy Sciences and Functional Foods, Faculty of Food Science and Nutrition, Poznań University of Life Sciences, Wojska Polskiego 31, 60-624 Poznań, Poland; joanna.kobus-cisowska@up.poznan.pl; 7 Institute of Molecular Physics, Polish Academy of Sciences, Smoluchowskiego 17, 60-179 Poznan, Poland; kornelia.lewandowska@ifmpan.poznan.pl; 8 Department of Biopharmacy, Faculty of Pharmacy, Medical University of Lublin, Chodzki 4a, 20-093 Lublin, Poland; anita.plazinska@umlub.pl; 9 Jerzy Haber Institute of Catalysis and Surface Chemistry, Polish Academy of Sciences, Niezapominajek 8, 30-239 Krakow, Poland; wojtek_plazinski@o2.pl

**Keywords:** piperine, hydroxypropyl-β-cyclodextrin, solubility, permeability, anti-inflammatory and neuroprotective activity

## Abstract

Piperine is an alkaloid that has extensive pharmacological activity and impacts other active substances bioavailability due to inhibition of CYP450 enzymes, stimulation of amino acid transporters and *P*-glycoprotein inhibition. Low solubility and the associated low bioavailability of piperine limit its potential. The combination of piperine with 2-hydroxypropyl-β-cyclodextrin (HP-β-CD) causes a significant increase in its solubility and, consequently, an increase in permeability through gastrointestinal tract membranes and the blood–brain barrier. X-ray powder diffraction (XRPD), differential scanning calorimetry (DSC), Fourier-transform infrared spectroscopy (FT-IR), nuclear magnetic resonance (NMR) were used to characterize interactions between piperine and HP-β-CD. The observed physicochemical changes should be combined with the process of piperine and CD system formation. Importantly, with an increase in solubility and permeability of piperine as a result of interaction with CD, it was proven to maintain its biological activity concerning the antioxidant potential (2,2-diphenyl-1-picryl-hydrazyl-hydrate assay), inhibition of enzymes essential for the inflammatory process and for neurodegenerative changes (hyaluronidase, acetylcholinesterase, butyrylcholinesterase).

## 1. Introduction

Piperine is an alkaloid found in plants of the species *Piper* in the family Piperaceae. Piperine exhibits a wide range of biological activity: anti-inflammatory, antioxidant, antibacterial, anticancer, immunomodulatory, antidiarrheal, analgesic, hepatoprotective, and neuroprotective in the central nervous system. It has anti-epileptic activity, cognitive-enhancing effect. It reduces malonaldehyde level and lipid peroxidation. It inhibits traumatic brain injury-induced seizures in mice [1]. Despite the proven multidirectional effects of piperine, its use is limited by low water solubility (40 mg/L at 18 °C) [2]. Piperine alleviates the bioavailability of other active substances according to inhibition of metabolizing CYP450 enzymes, particularly CYP3A4, CYP2C9, and CYP1A2, stimulation of amino acid transporters in the lining of the intestine, and inhibition of *P*-glycoprotein [3,4]. However, piperine itself has poor bioavailability.

Studies confirm that the improvement of piperine’s solubility influences its higher bioavailability and increases the potential of pharmacological activity. Hitherto, it has been proven that combinations with selected excipients help to improve the solubility of piperine. E. Zaini et al. and Yueyi Deng et al. used the slurry method and the solvent evaporation method to prepare piperine systems with succinic acid and hydroxypropylmethylcellulose acetate succinate, respectively [5,6]. By using the above-mentioned techniques, multicomponent crystal and amorphous solid dispersion were obtained, which were characterized by single-crystal X-ray diffraction analysis (XRPD), differential scanning calorimetry (DSC), Fourier-transform infrared spectroscopy (FT-IR). For all tested combinations, an increase in solubility of about four times was proved. For amorphous solid dispersion, the increase in apparent permeability was also determined. According to Tianjing Ren et al. and Diana Anissian et al. preformulation studies of piperine, an increase in biological activity was also obtained by preparing piperine-loaded nanoparticles and piperine-loaded chitosan–sodium tripolyphosphate nanoparticles, respectively. There was an increase in bioavailability of piperine with a promising anti-epileptic effect and enhanced neuroprotection and amelioration of the astrocytes activation in the chemical kindling model of epilepsy.

In order to impact the physicochemical and biological properties of piperine, it was also combined with cyclodextrins (CDs). T. Esau et al. prepared a co-ground mixture of piperine and β-CD in a molar ratio 1:1. The creation of the inclusion complex was investigated with DSC, XRPD, Raman, and nuclear magnetic resonance (NMR) spectroscopy. The studies confirmed the formation of an inclusion complex in a ratio of 1:1, which increased the solubility of piperine in dissolution testing [7]. Inclusion complexes have also been obtained by Bruno N Teixeira et al. for black pepper oleoresin with hydroxypropyl beta-CD (HP-β-CD) by kneading technique and by M. Quilaqueo et al. for piperine with β-CD by freeze-drying technique [8,9]. In all the tested systems, it was proven that those system preparation methods induce an increase in biological activity—the antioxidant capacity and antimicrobial activity. For black pepper oleoresin, it was impossible to achieve a sufficient pungent flavor masking, but with an appropriate ratio and preparation technique of the systems of piperine and CDs, it is possible to mask the taste of the alkaloid.

Considering the effects of piperine interaction with CDs, this study aimed to evaluate the effect of combining piperine with HP-β-CD using the simplest technique—kneading. The effect of combining piperine with HP-β-CD was studied concerning changes in solubility and permeability simulating the permeability through the gastrointestinal tract (GIT) and the blood–brain barrier (BBB), and biological properties: antioxidant activity ((2,2-diphenyl-1-picryl-hydrazyl-hydrate (DPPH) assay), possible inhibition of inflammation-induced neurodegenerative enzymes (hyaluronidase, acetylcholinesterase (AChE) and butyrylcholinesterase (BuChE) inhibition).

## 2. Results and Discussion

Black pepper, a spice used every day in kitchens worldwide, is rich in piperine. Piperine exhibits many biological effects and affects the pharmacokinetics of drugs and other substances taken in along with the nourishment. The limitation in using the biological activity of piperine is its low solubility. Thus, increasing the solubility of piperine will increase its bioavailability and, consequently, an improvement in biological activity. Cyclodextrins are well-known enhancers of solubility, stability, and permeability. Moreover, they positively impact body weight, serum lipid control and have prebiotic effects [10]. Therefore, it is worth modifying the physicochemical properties of a poorly soluble substance, which may have even wider application according to the combination with HP-β-CD. According to the European Medicines Agency (EMA/CHMP/495747/2013), HP-β-CD is approved for use in oral products and is found to be generally safe. As an oral pharmaceutical, even 8000 mg/day of HP-β-CD can be taken orally [11]. FDA cites HP-β-CD on the list of inactive pharmaceutical ingredients, and the USP/NF and EP reference HP-β-CD [12,13]. The use of HP-β-CD as a spice additive is highly justified.

Bearing in mind the possible modifications of piperine due to interaction with cyclodextrins, in this work, optimization studies of the interaction of piperine with cyclodextrin were carried out. The piperine-cyclodextrin system was obtained, and the activity of piperine in the cyclodextrin system was assessed. The values determined for free piperine were taken as reference values.

**Figure 1:**
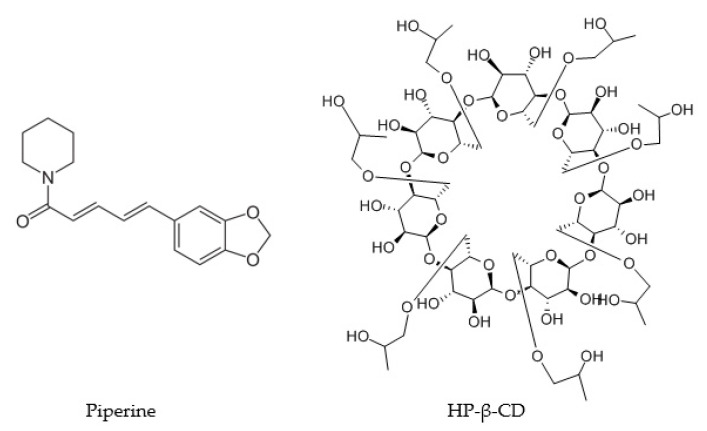
The structure of piperine and combination of piperine with 2-hydroxypropyl-β-cyclodextrin (HP-β-CD).

In the beginning, theoretical analysis was carried out, which enabled the assessment of the formation of systems of piperine and HP-β-CD (the structures are shown in Figure 1). The binding energies found during docking simulations differ, depending on the accepted HP-β-CD structure. The energies determined for the DFT structure vary in the range of −5.5–5.0 kcal/mol, whereas that characteristic of the UFF-derived structure fall in a much narrower range of −6.0–5.9 kcal/mol. Both the negative sign of the determined values and their magnitudes suggest a strongly favorable binding mode.

**Figure 2:**
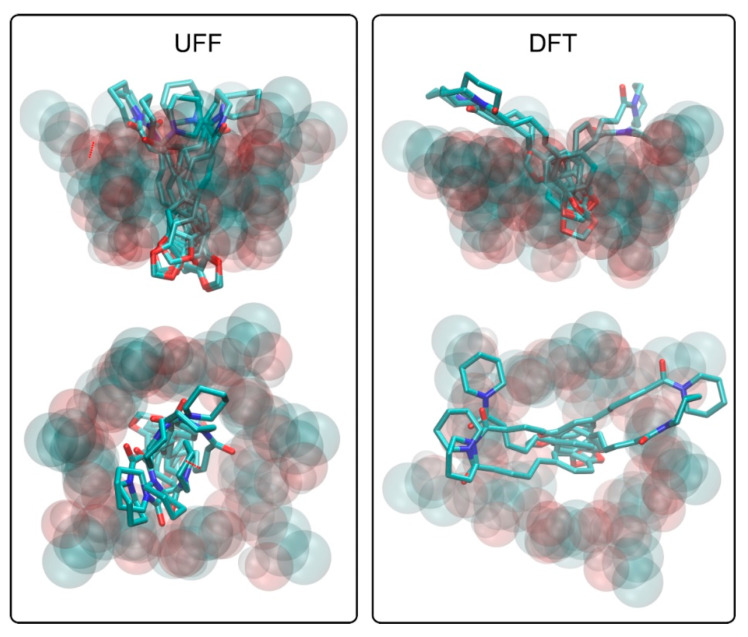
The piperine–HP-β-CD complexes obtained in the study: UFF-optimized structure of HP-β-CD (**left**) and density functional theory (DFT)-optimized structure of HP-β-CD (**right**). See the main text for details.

The analysis of the piperine–HP-β-CD systems provides an insight into the qualitative pattern of interactions that may be the driving force for binding as well as into the preferred spatial arrangement of the bound ligand molecule in the hosting cavity. The graphical illustration of the considered structures is given in Figure 2. Due to marginal differences in binding energies, all identified ligand poses are shown, and the discussion below concerns the collective set of various complexes rather than individual poses. This is also justified because the extremely similar values of binding energies are correlated with relatively similar sets of binding patterns. However, the two main pattern types can be distinguished depending on the accepted HP-β-CD structure.

The UFF-optimized structure exhibits a more compact conformation, with the HP moieties located close to each other and forming an extension of the inner CD-binding cavity. The ligand’s piperidine ring interacts with the exocyclic HP chains through hydrophobic contacts between respective aliphatic parts of those two groups. The attractive forces resulting from those contacts are additionally enhanced by the accompanying hydrogen bonding between hydroxyl groups of CD and the carbonyl oxygen atom of piperine. The rest of the piperine molecule is immersed into the inner cavity of CD; the corresponding intermolecular contacts involve the aliphatic patches of glucose residues and the piperine molecule’s central chain, which encompasses multiple unsaturated bonds. The involved interactions are probably of the CH-π type, characteristic of the carbohydrate-binding by other biomolecules [14]. The second, benzodioxole ring of piperine, interacts by hydrogen bonding with hydroxyl groups of HP-β-CD, located at the second edge of the inner cavity of CD.

The second, DFT-optimized structure of HP-β-CD exhibits a looser packing of HP sidechains and a more extended entry to the inner binding cavity. This is reflected by slightly different piperine-binding modes obtained in this case. The piperidine moiety of the ligand still interacts with the exocyclic HP chains through roughly the same pattern of contacts. However, due to different orientations of those chains, the central part of the piperine molecule is shifted toward the main entrance to the binding cavity. This disrupts the contacts of hydroxyl groups of CD with benzodioxole ring and creates the CH-π interactions with glucose rings instead. At the same time, the interactions of the central piperine chain with residues of the inner cavity are significantly reduced. The total energy balance of such change in the configuration in relation to the UFF structure is unfavorable, as can be deduced from the calculated binding energies.

Judging from the other studies in which CDs exhibit significant molecular flexibility as well as from the current results according to which several energetically equivalent structures were found, we speculate that the actual binding mode may be a superposition of numerous configurations of both piperine molecule in the binding cavity as well as the hosting HP-β-CD molecule. The molecular fragments of the complex that are most likely to undergo the conformational rearrangements are the HP moieties attached to CDs. The main driving force for binding is a combination of the hydrophobic interactions involving piperidine moiety and HP groups, supported by the CH-π interactions between hydrophobic patches of glucose residues located in the inner CD cavity and either benzodioxole ring or the central, unsaturated chain of the ligand.

**Figure 3:**
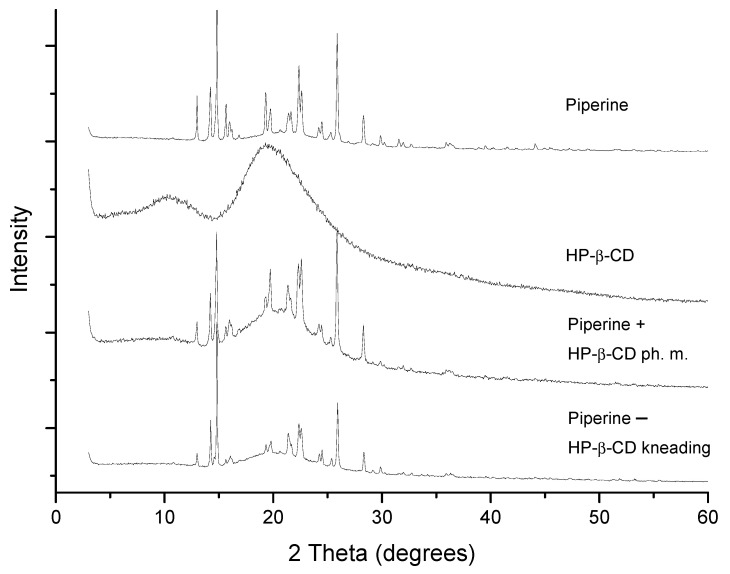
X-ray powder diffraction (XRPD) diffraction patterns of piperine, the mixture with HP-β-CD obtained by kneading method and physical mixture.

**Figure 4:**
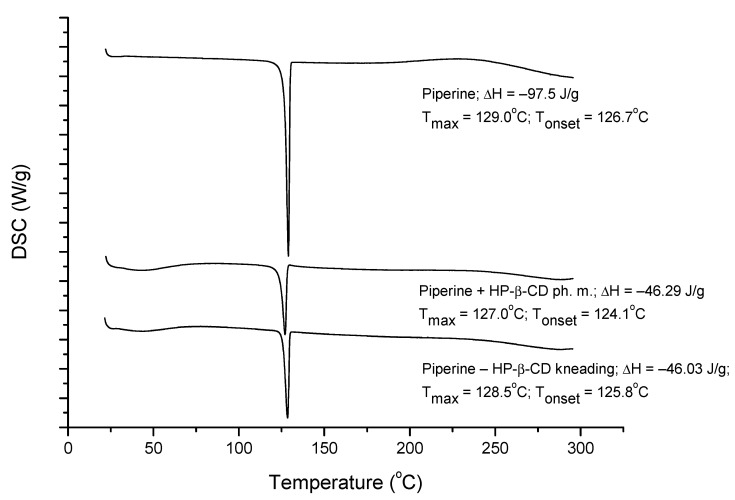
Differential scanning calorimetry (DSC) thermograms of piperine, the mixture with HP-β-CD obtained by kneading method and physical mixture.

The combinations of piperine and CD were characterized by the techniques: XRPD, DSC, NMR, and FT-IR. XRPD patterns are shown in Figure 3. HP-β-CD diffractogram is typical for an amorphous material. Piperine produces a crystalline pattern consisting of a series of well-defined sharp peaks. Superimposed diffraction patterns were obtained for HP-β-CD + piperine physical mixture and two-component sample prepared by kneading. Diffractometric and thermal analysis (Figure 4) allowed to exclude a strong piperine and HP-β-CD complex formation. However, the formation of a weak complex between piperine and HP-β-CD can be suggested. The kneading technique did not require any energy input, only a minimum amount of solvents. Thus, based on XRPD and DSC methods, the possibility of only intermolecular piperine–CD interaction via piperine adhesion on the CD surface or a weak inclusion of piperine into HP-β-CD can be suggested.

The formation of piperine systems with HP-β-CD does not require the expenditure of energy. It can occur spontaneously, for example, during the processing of black pepper with the CD since CDs are effective extracting compounds. El Darra N. et al. study proved the functionality of cyclodextrins as extracting agents. CD-assisted extraction of polyphenols from peach pomace was compared with extraction with an organic solvent—ethanol in a solid–liquid ratio of 1:10 (*w/v*). The same concentrations were used for both β-CD and ethanol extraction (10 mg/mL, 20 mg/mL, 30 mg/mL, 40 mg/mL, and 50 mg/mL). Extracts obtained in β-CD assisted extraction compared to organic solvent extraction were superior in total polyphenol content and resulted in stronger antiradical and antibacterial activity [15]. In another study, β-CD was used in water and ethanolic ultrasound-assisted extraction of Red beet compounds (1:10 *w/v*). Extracts obtained with 5% β-CD solutions showed the highest betanin content, phenolic compounds, and the strongest antioxidant activity. After using β-CD-enhanced ultrasound-assisted extraction, the Red beet active compounds were more stable than after extraction of pure water or ethanol [16].

**Figure 5:**
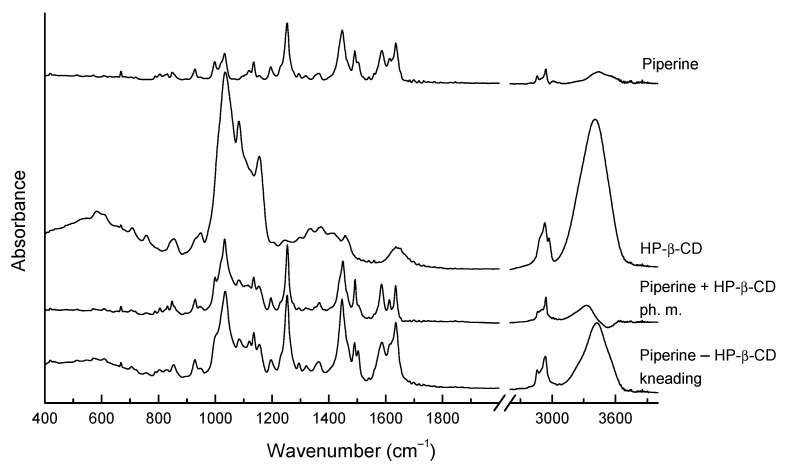
The experimental FT-IR of piperine, HP-β-CD, the mixture with HP-β-CD obtained by kneading method and physical mixture.

IR spectrum of the physical mixture and two-component sample prepared by kneading technique of piperine and HP-β-CD appear similar (Figure 5). Thus, it can be concluded that various techniques for obtaining the piperine and HP-β-CD structure gave a system with identical interactions between components. Nevertheless, some changes are visible in the spectra of the piperine–HP-β-CD mixture and pure components. The biggest changes are observed in the range 900–1200 cm^−1^, where strong bands from the HP-β-CD are located. A different character of the bands is visible; their shapes are changed and, to a small extent, also the position of the bands. The bands related to the piperine’s vibration located at 928, 999, 1031, 1118, 1135, 1154, 1197 cm^−1^ for piperine–HP-β-CD are shifted to 927, 989, 1032, 1120, 1136, 1153 and 1194 cm^−1^, respectively. For the piperine–HP-β-CD is also visible the band at 1084 cm^−1^ related to the stretching vibration of the C—C, C—O bonds, and wagging vibration of the C—H bonds directly at the sugar ring at C_4_ and C_5_ carbon in the HP-β-CD. At higher frequencies, also slight shifts of bands are visible, and, for example, the bands for piperine located at 1448, 1490, 1584, 1614 and 1636 cm^−1^, for piperine–HP-β-CD are shifted to 1446/1450 (kneading/physical mixture), 1490, 1586, 1613 and 1637 cm^−1^. Despite such small differences compared to those described in the works of Quilaqueo et al. Ezawa et al., one can assume that a weak complex between piperine and HP-β-CD is formed strong shifts of bands are visible in the range above 3000 cm^−1^ where are located the bands related to the stretching vibration of the O–H bonds [9,17]. For the HP-β-CD, the band is located at 3400 cm^−1,^ and for piperine and HP-β-CD is shifted to 3330 and 3423 cm^−1^ for physical mixture and structure obtained by kneading respectively. This suggests the formation of hydrogen bonds between piperine and HP-β-CD. The same can be assumed that the methylenedioxyphenyl ring will move into the CD’s cavity and forms an OH bond with the hydroxyl group of the HP-β-CD. When the ratio I1636/I1584 was calculated, the value was found to be 1.201 for piperine. However, for the piperine + HP-β-CD (physical mixture) and for the piperine–HP-β-CD (kneading), the ratios of I1636 to I1584 were 0.973 and 1.232, respectively. The decrease in intensity of C=C stretching modes of aromatic ring motifs of piperine in the complex obtained by kneading suggests the presence of a surrounding obstacle opposing the IR active vibrational mode. The reduction in intensity is apparently due to the shielding of the methylenedioxyphenyl ring under the influence of the HP-β-CD cavity.

**Figure 6:**
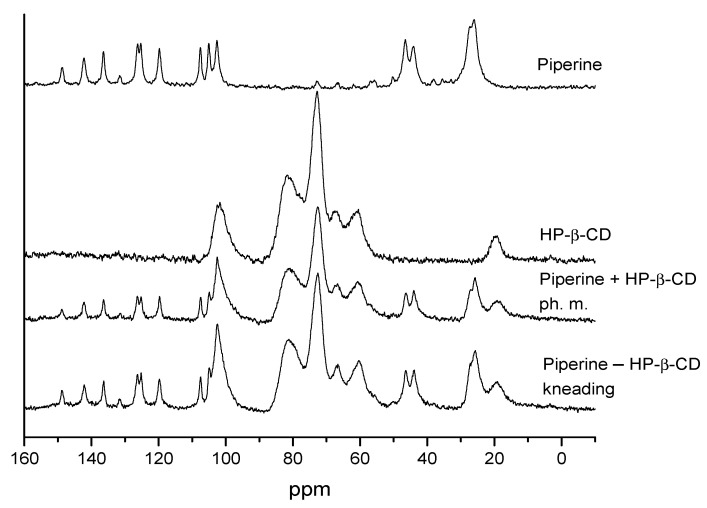
^13^C NMR spectra of piperine, HP-β-CD, physically mixed compounds, and kneaded compounds.

As shown in Figure 6, the solid-state ^13^C NMR spectra recorded for the physical mixture and the kneading mixture are virtually indistinguishable, and they both represent a simple superposition of spectra acquired for individual components, i.e., CD and piperine. It is worth emphasizing that all NMR signal parameters, namely their positions and linewidths, are preserved after mixing. This result suggests that if there is any interaction taking place between two components, it must be subtle since it does not alter the chemical environment of detected carbons substantially, i.e., chemical shielding is preserved.

**Figure 7:**
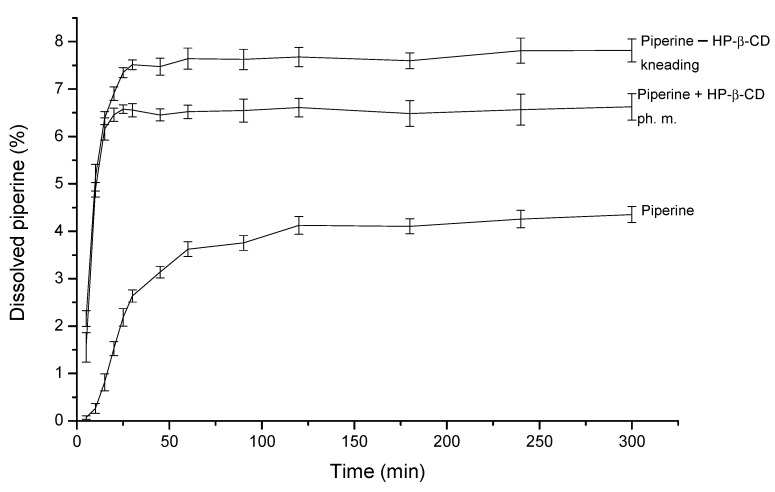
Apparent solubility of piperine, the mixture with HP-β-CD obtained by kneading method and physical mixture.

The occurrence of interactions between poorly soluble molecules and hydrophilic macromolecules may result in an increase in their solubility. The dissolution study for combinations of piperine and cyclodextrins (Figure 7) was conducted for 5 h, the plateau is achieved for pure piperine after 120 min, while piperine in kneading the mixture with CD reaches the plateau in about 30 min, which proves the increase in the dissolution rate of piperine in the kneading mixture. The maximum value for piperine in the kneading mixture was 7.82 ± 0.24%, while for dissolved piperine, it was 4.35 ± 0.17% (solubility of piperine in the physical mixture was 6.62 ± 0.28%). The solubility of the piperine in the kneading mixture was almost doubled comparing to pure piperine, which is statistically significant according to fit factor f_1_. The percentage of dissolved piperine in every time point was as follows: kneading mixture > physical mixture > pure piperine.

**Figure 8:**
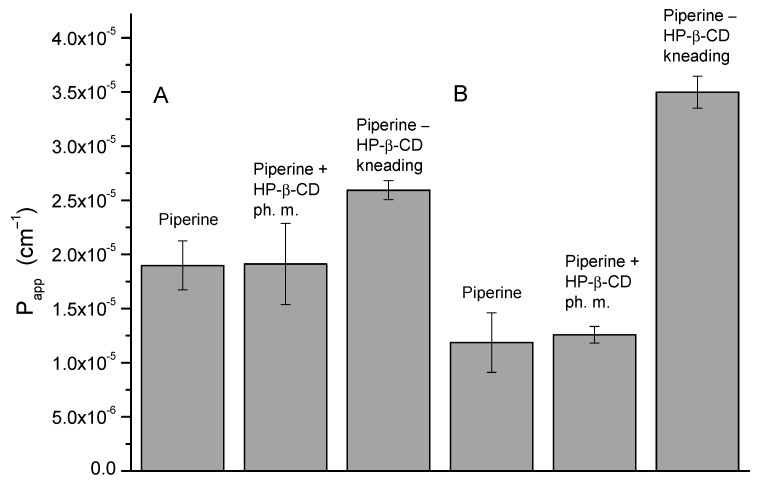
Values of apparent permeability coefficients of piperine determined for gastrointestinal tract (GIT) permeability (**A**) and permeability through the BBB (**B**).

The consequence of the enhanced solubility of piperine was its greater permeability through the system of artificial biological membranes. The study of permeability through the mixture of artificial biological membranes was carried out in the GIT (pH ≈ 6.8) and BBB (pH ≈ 7.4) conditions; the results are shown in Figure 8. In the study for the GIT, the P_app_ value for piperine is 1.90 × 10^−5^ ± 2.25 × 10^−6^ cm/s, for the physical mixture, it is 1.91 × 10^−5^ ± 3.74 × 10^−6^ cm/s, and for the kneading, the mixture is 2.59 × 10^−5^ ± 8.76 × 10^−7^ cm/s. The 1.36-fold increase in the permeability of piperine might elevate the bioavailability in vivo after oral administration. Whereas in the PAMPA BBB test, there is an almost threefold increase in permeability of piperine across the BBB, which increases the potential for piperine to act within the central nervous system, for instance, as a neuroprotective agent (the P_app_ value for piperine is 1.19 × 10^−5^ ± 2.75 × 10^−6^ cm/s, for the physical mixture it is 1.26 × 10^−5^ ± 7.65 × 10^−7^ cm/s, and for the kneading, the mixture is 3.50 × 10^−5^ ± 1.48 × 10^−6^ cm/s).

Many articles describe the effect of piperine on the increase in other substances’ permeability, such as linarin or curcumin, which was studied in rat jejunum segment using in situ single-pass perfusion technique or ex vivo using non-everted intestinal sac [18,19]. Piperine also increases the bioavailability of the substances [20,21]. For the more complex piperine-CD systems, where additionally curcumin was added, an improvement in important physicochemical properties was also noted to improve their bioavailability [4]. The modification of silybin bioavailability with strong hepatoprotective potential, but the use limited by low bioavailability was studied by Xiaoli Bi et al. A combination of silybin (50 mg/kg) and piperine (50 mg/kg) was administered to rats. The bioavailability was enlarged 146% for total silybin A and 181% for total silybin B. Piperine increased the absorption of silybin according to inhibition of the efflux transporters, including multidrug resistance-associated protein 2 and breast cancer resistance protein [20]. According to the Singha A. study, piperine (10 mg/kg) administered to rats with atenolol (100 mg/kg) increased the bioavailability of active pharmaceutical ingredients [21].

On the other hand, the number of publications modulating the permeability of piperine is much smaller. Eman A. Ashour et al. prepared formulations of 10% *w*/*w* piperine/Soluplus^®^ using hot-melt extrusion [22]. The authors obtained an increase in in vitro release and enhanced piperine permeability to 158.9 μg/5 mL tested using the Caco-2 model. After oral administration, piperine reaches the brain effectively at the dose of 35 mg/kg [23]. According to the Caco-2 monolayer model, the permeability coefficients were 5.41 × 10^−5^ cm/s for basolateral-to-apical transport and 4.78 × 10^−5^ cm/s for apical-to-basolateral transport [23]. Eigenmann D. E. et al. investigated the blood–brain permeability of piperine and its analogs in three in vitro models: a human model with immortalized hBMEC cells, a human brain-like endothelial cells model, and a primary animal model. Based on both studies’ efflux ratios, it can be concluded that piperine is not much involved in active transport [23,24]. However, there are no preformulation studies that would cause modifications to piperine’s permeability across the blood–brain barrier beyond our previous studies in combination with curcumin and cyclodextrin [4].

The DPPH antioxidant study was carried out using vitamin C as a reference (IC_50_ = 0.066 mg/mL). The IC_50_ value for piperine was 38.95 ± 1.84 mg/mL; for the kneading mixture, it was 37.18 ± 0.86 mg/mL. For the physical mixture, it was 38.43 ± 0.94 mg/mL. The piperine antioxidant activity after interaction with CD remained at the same level.

The anti-hyaluronidase activity assay showed that the IC_50_ value for pure piperine (13.26 mg/mL) increased to 14.06 mg/mL in the kneading mixture, and for the physical mixture decreased to 13.14 mg/mL. The preparation of the physical mixture of the piperine with HP-β-CD increased the inhibition of hyaluronidase activity by piperine.

**Figure 9:**
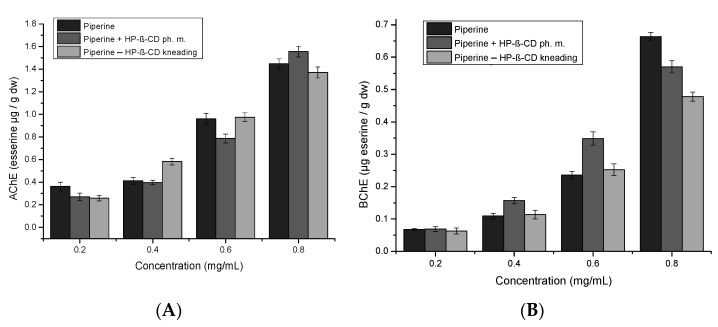
The activity of piperine, piperine mixture with HP-β-CD and physical mixture as AChE (**A**) and as BuChE inhibitors (**B**).

The inhibition of AChE and BuChE (Figure 9) increases with the concentration of the piperine. The highest inhibition of AChE is at the concentration of 0.8 mg/mL piperine in a physical mixture with HP-β-CD (1.56 ± 7.8 × 10^−2^ esserine μg/g dw), while BuChE for pure piperine (0.66 ± 1.3 × 10^−2^ esserine μg/g dw). For concentrations of 0.4 mg/mL and 0.6 mg/mL, the highest inhibition of AChE occurs for kneading mixture (0.58 ± 2.913 × 10^−2^ esserine μg/g dw) and (0.97 ± 4.9 × 10^−2^ esserine μg/g dw), respectively. The inhibition of BuChE 0.4 mg/mL and 0.6 mg/mL is the highest for physical (0.16 ± 9.9 × 10^−3^ esserine μg/g dw) and (0.35 ± 2.0 × 10^−2^ esserine μg/g dw), respectively. An elevation in the solubility of a substance having butyrylcholinesterase inhibition activity may result in an increase in the inhibition of this enzyme, as was already noted in the literature. According to Songngam S. et al.’s study, 5,7-dimethoxyflavone isolated from *Kaempferia parviflora* was combined with HP-β-CD by the freeze-drying method in a 1:1 molar ratio [25]. The solubility of 5,7-dimethoxyflavone after complexation was 361.8-fold enlarged, and the inhibitory activity of butyrylcholinesterase in vitro (in terms of the IC_50_ value) increased 2.7 times.

## 3. Materials and Methods

### 3.1. Materials

Piperine with a purity > 97% and HP-β-CD (molar substitution 0.8) were purchased from Sigma-Aldrich (Poznan, Poland). Acetonitrile (high-performance liquid chromatography (HPLC) grade) was obtained from Merck KGaA (Darmstadt, Germany). Acetic acid, methanol (HPLC grade), dimethyl sulfoxide (HPLC grade), sodium chloride, and potassium dihydrogen phosphate were provided by Avantor Performance Materials (Gliwice, Poland). Prisma HT, GIT lipid solution, Acceptor sink buffer was purchased from Pion Inc. (Forest Row, UK).

### 3.2. Theoretical Studies

The piperine ligand molecule was prepared by using the Avogadro 1.1.1 software [26] and optimized within the Universal force field (UFF) [27] (5000 steps, steepest descent algorithm). The structure of HP-β-CD was prepared based on the available crystal structure of unfunctionalized β-CDs, the results of our earlier (unpublished) studies as well as the structural parameters available in the literature [28]. According to the experimental data, the molar weight of HP-β-CD corresponds to a unique substitution pattern, according to which only a single glucose residue lacks unfunctionalization. The hydroxymethyl groups attached to HP-β-CD were initially oriented in the trans conformation, in agreement with the dominated conformer found in glucose monosaccharide. The 2-HP moieties were oriented in order to facilitate the hydrogen bonding-mediated contacts between neighboring units. The resulting structure was optimized within the UFF force field (10,000 steps) and either directly subjected to docking or further optimized at the high-level theory (DFT/B3LYP/6-311G (d,p) [29]. The flexible, optimized ligand molecule was docked into those two structures of HP-β-CD (denoted, following the optimization level, as UFF and DFT). The AutoDock Vina 4.0 software [30] was applied for docking simulations. The docking procedure was carried out within the cuboid region large enough to accommodate the whole HP-β-CD molecule. All the default procedures and algorithms implemented in AutoDock Vina were applied during the docking procedure. The complexes were analyzed in terms of binding energies and binding-related interactions.

### 3.3. Preparation of Piperine–HP-β-CD Mixture

The piperine mixture ––HP-β-CD in the mass ratio 1:1—was prepared according to the kneading technique. Both substances were ground in the mortar. Next, both substances, with a minor amount of alcohol and distilled water used to receive a pasty consistency, were ground for 60 min in mortar. Later, it was drying at 45 °C for 24 h, and then the mixture was ground again for 10 min. The physical mixture in the same ratio of the compounds was also prepared.

### 3.4. Characterization of Piperine–HP-β-CD Mixture

XRPD, DSC, FT-IR spectroscopy, and NMR spectroscopy were used to examine the piperine and HP-β-CD solid-state interactions.

#### 3.4.1. XRPD

The characterization of the prepared mixtures was carried out by the XRPD method. Diffraction patterns were recorded on a PANalytical Empyrean diffractometer (Malvern Panalytical, Malvern, UK) with CuK_α_ radiation (1.54056 Å) at a tube voltage of 45 kV and a tube current of 40 mA. The angular range was 3° to 50° with a step size of 0.017° and a counting rate of 15 s/step. The analysis of the acquired data was performed using OriginPro 8 [31].

#### 3.4.2. DSC

Thermal analysis was performed using DSC 204 Phoenix differential scanning calorimeter (Netzsch, Selb, Germany). Then, 5 mg powdered samples were placed in hermetically enclosed aluminum cells next to their as the reference sample and heated at a scanning rate of 5 K min^−1^ from 294 to 295 °C in a helium atmosphere with a flow rate of 40 mL min^−1^. The experimental procedure and data processing details were identical to those described by us for other pharmaceutical compounds [4,32].

#### 3.4.3. FT-IR Spectroscopy and Density Functional Theory (DFT) Calculations

Infrared spectra were performed on an FT-IR Bruker Equinox 55 spectrometer (Bruker, Billerica, MA, USA). In the hydraulic press, the substances, the mixture, and the physical mixture were turned into pellets with IR grade KBr. Then the spectra were recorded in a frequency range of 400–4000 cm^−1^. As supportive methods, the DFT calculations with a B3LYP functional and 6–31G(d,p) as a basis set were used.

#### 3.4.4. NMR Analysis

Cross-polarization (CP) pulse sequence with magic angle spinning (MAS) and dipolar decoupling of protons was applied. Samples were placed into a 4 mm diameter zirconia rotor and spun with 7 kHz. ^13^C spectrum collected was a result of NS = 4000 accumulations.

### 3.5. Apparent Solubility and Permeability through Membranes Simulating GIT Walls and BBB of the Piperine–HP-β-CD Mixture

Alternations in concentrations of piperine during solubility study were measured by HPLC with the diode-array detector (DAD). The piperine determination in apparent solubility study was carried out using a Phenomenex-C18 column (250 mm × 4.6 mm; 5 µm) as the stationary phase and methanol and water (80:20, *v/v*) mixture as the mobile phase. The column temperature was set at 30 °C. The detection wavelength was set at 343 nm, and the flow rate of the mobile phase was 1.4 mL/min. The analysis was performed with the injection volume set at 100.0 µL. The differences in concentrations of piperine during permeability studies were recorded spectrophotometrically on a plate reader at 343 nm.

The dissolution study was carried out in the paddle apparatus. Piperine and the mixture with CD (containing 4 mg of piperine) were weighed to gelatin capsules, which were later implemented to springs to sink and prevent flotation on the surface of the medium. The study was carried out at a pH of 6.8 in sink conditions. The vessels were filled with 500 mL of phosphate buffer, the temperature was maintained at 37 °C, the paddles were set at the stirring speed of 100 rotations per minute. At predetermined time points, 5.0 mL samples were withdrawn and replaced with equal volumes of temperature-equilibrated media and filtered through a membrane filter (0.45 μm). The dissolution profiles were compared using two-factor values *f*_1_ and *f*_2_ implemented by Moore and Flanner [33] according to the equations:

$$f_{1}=\frac{\sum_{j=1}^{n}\left|R_{j}-T_{j}\right|}{\sum_{j=1}^{n}R_{j}}\times 100$$

$$f_{2}=50\times \log\left({\left(1+\left(\frac{1}{n}\right)\overset{n}{\underset{j=1}{\sum }}{\left|R_{j}-T_{j}\right|}^{2}\right)}^{-\frac{1}{2}}\times 100\right)$$

where *n* is the number of time points, *R_j_* is the percentage of the reference dissolved substance in the medium, *T_j_* is the percentage of the dissolved tested substance, *t* is the time point. Dissolution profiles are described as similar when the *f*_1_ value is close to 0, or *f*_2_ is close to 100 (between 50 and 100) [33].

In vitro GIT and BBB permeability was performed using the parallel artificial membrane permeability assay model (PAMPA). The sandwich consists of two 96-well microfilter plates. The PAMPA mixture contains two chambers: the donor at the bottom and the acceptor chamber at the top. The chambers are separated by a 120 μm-thick microfilter disc coated with a 20% (*w/v*) dodecane solution of a lecithin mixture (Pion, Inc.). The samples were dissolved in dimethyl sulfoxide (baseline concentration of piperine in the samples—5.0 mg/mL) and later added to the donor compartments. The donor solution was adjusted to pH ≈ 6.8 for GIT application and to pH ≈ 7.4 for BBB application, using 0.5 M NaOH. The chambers were combined and then incubated for 3 h for the GIT model and 4 h for the BBB model in a humidity-saturated atmosphere at the temperature set at 37 °C. The apparent permeability coefficient (*P_app_*) was calculated according to the following equation:

$$P_{app}=\frac{-ln\left(1-\frac{C_{A}}{C_{equilibrium}}\right)}{S\times \left(\frac{1}{V_{D}}+\frac{1}{V_{A}}\right)\times t}$$

where *V_D_*—donor volume, *V_A_*—acceptor volume, *C_equilibrium_*—equilibrium concentration $C_{equilibrium}=\frac{C_{D}\times V_{D}+C_{A}\times V_{A}}{V_{D}+V_{A}}$, *S*—membrane area, *t*—incubation time (in seconds). Substances with the value of *P_app_* in the GIT model below 0.1 × 10^−6^ cm/s are described as low permeable, compounds found as medium permeable have a 0.1 × 10^−6^ cm/s ≤ *P_app_* < 1 × 10^−6^ cm/s and compounds with a *P_app_* ≥ 1 × 10^−6^ cm/s are defined as ones with high permeability [34]. Substances whose *P_app_* in BBB model is <2.0 × 10^−6^ cm/s are described as low permeable. Compounds with the *P_app_* value in the range of 2.0 to 4.0 × 10^−6^ cm/s are defined as substances with questionable permeability. Compounds with high permeability present the *P_app_* value at the level > 4.0 × 10^−6^ cm/s [35].

### 3.6. Biological Activity

#### 3.6.1. Antioxidant Activity

The antioxidant properties were analyzed by spectrophotometric DPPH assay, according to Studzińska-Sroka’s modifications [36]. Methanol solution of DPPH (0.2 mM) and rising concentrations of piperine (4.4–44.0 mg/mL) alone and in the mixture with HP-β-CD were prepared. 25.0 µL of DPPH solution was mixed with 175.0 µL of studied solutions in a 96-well plate. The plate was shaken and incubated in darkness for 30 min at room temperature. Afterward, the absorbance (*A*) was measured at the wavelength set at 517 nm against the blank (the mixture of DPPH solution and methanol). The inhibition of the DPPH radical by the studied samples was calculated due to the following equation:

$$A=\frac{A_{o\;}-A_{i}}{A_{o}}\times 100\%$$

where *A_o_* is the control sample absorbance, *A_i_* is the absorbance of the studied sample. Every determination was repeated six times. As a reference, vitamin C (in concentrations 10–100 µg/mL) was used. Half maximal inhibitory concentration (IC_50_) values, which determine the substance concentration that inhibits the formation of DPPH by 50%, were determined by linear regression analysis.

#### 3.6.2. Determination of Hyaluronidase Inhibition

The hyaluronidase inhibition was evaluated using the turbidimetric method’s Grabowska with slight changes [37]. The study was performed in the 96-well filter plate. 25.0 µL of incubation buffer, 25.0 µL of hyaluronidase solution, 10.0 µL of the sample (the concentrations of piperine 10–20 mg/mL) and 15.0 µL of acetate buffer (pH 4.5) was added to the plate. Subsequently, the plate was kept for 15 min in the temperature of 310 K. After this time, hyaluronic acid (25.0 μL) was added to the wells, and the plate was incubated for 45 min at 310 K. Finally, 200.0 µL of hexadecyltrimethylammonium bromide solution was added, and cells were incubated for 10 min at room temperature (20 °C). The turbidity was measured spectrophotometrically at 600 nm. Each test was repeated five times. The inhibition of hyaluronidase was calculated from the equation:

$$I\%=\frac{\left(P-B_{3}\right)-\left(B_{2}-B_{1}\right)}{\left(B_{4}-B_{3}\right)-\left(B_{2}-B_{1}\right)}\times 100\%$$

where *P*—sample turbidity, *B*_1_—absorbance of blank 1 (blind control), *B*_2_—absorbance of blank 2 (with enzyme and hyaluronic acid—determination of enzyme properties), *B*_3_—absorbance of blank 3 (investigating tested substances and mixtures absorbance at 600 nm with the enzyme), *B*_4_—absorbance of blank 4—(investigating tested substances’ and mixture’s absorbance at 600 nm with the hyaluronic acid).

#### 3.6.3. Determination of AChE and BuChE Inhibition

The modified spectrometric method developed by Ellman et al. and described by Kobus-Cisowska et al., was used to measure the activity of piperine and the mixture with CD [38,39]. A POLARstar Omega (BMG LABTECH, Ortenberg Germany) plate reader was used for measurements of 96-well plates of the maximum volume of 300 μL. Acetylthiocholine/butyrylthiocholine hydrolysis caused a change of color. The absorbance of the enzymes was measured at a wavelength of 412 nm, ten minutes after pipetting on a microplate. The reaction mixture containing 0.1 mL of 0.3 mM 5,5-dithio-bis-(2-nitrobenzoic acid) (DTNB, Sigma-Aldrich, Schnelldorf, Germany), 10 mM NaCl and 2 mM MgCl_2_•6H_2_O solution, 0.575 mL 50 mM Tris-HCl buffer (pH = 8.0), 25 μL of 0.28 units/mL AChE/BuChE (Sigma-Aldrich, Germany) and 0.2 mL of tested extract was measured at a wavelength of 405 nm and at a temperature of 22 °C. The measurement was conducted after 20 min (BuChE) or 60 min (AChE) after adding all ingredients into a microplate. The blank sample contained Tris-HCl buffer instead of tested compounds. A positive–negative control was applied, and it consisted of 90.7 μM eserine instead of tested compounds. All samples were analyzed in eight independent replicates. The inhibition of each enzyme was calculated using a calibration curve. The calibration curves were prepared using serine as a standard at concentration ranges between 0.09 and 6.10 μM for AChE and 0.09–8.57 μM for BuChE.

## 4. Conclusions

Piperine is an alkaloid that exhibits multidirectional biological activity and, additionally, the possibility of inhibiting the activity of the cytochrome CYP450. The poor solubility of piperine is a significant limitation in using its biological activity. The fact, confirmed by our research results, of the interaction of piperine with cyclodextrins, is essential from the point of improving the solubility of piperine and masking its taste. Improvement of permeation through membranes simulating the gastrointestinal system walls and the blood–brain barrier are arguments for using improved inhibition of CYP450 activity and activities within the central nervous system. Changes in the biological activity of piperine resulting from interaction with cyclodextrins in most cases are correlated positively, which indicates that cyclodextrins should be treated as valuable reagents in obtaining valuable connections with piperine, not only in the area of technological and functional benefits.

## Data Availability

Data available in a publicly accessible repository.

## Abbreviations

AChE	Acetylcholinesterase
BBB	Blood–brain barrier
BuChE	Butyrylcholinesterase
CD	Cyclodextrin
DAD	Diode-array detector
DFT	Density functional theory
DPPH	2,2-Diphenyl-1-picryl-hydrazyl-hydrate
DSC	Differential scanning calorimetry
FT-IR	Fourier-transform infrared spectroscopy
GIT	Gastrointestinal tract
HPLC	High-performance liquid chromatography
HP	Hydroxypropyl
IC_50_	Half-maximal inhibitory concentration
MIC	Minimum inhibitory concentration
NMR	Nuclear magnetic resonance spectroscopy
PAMPA	Parallel artificial membrane permeability assay
*P_app_*	Apparent permeability coefficient
UFF	Universal force field
XRPD	X-ray powder diffraction
